# AI based ECG data recovery and cardiovascular diseases classification (CEDRC-network)

**DOI:** 10.1038/s41598-026-46232-3

**Published:** 2026-04-06

**Authors:** Muhammad Raheel Khan, Zunaib Maqsood Haider, Jawad Hussain, Masood Ahmad Khan, Saad Abdullah

**Affiliations:** 1 Department of Electrical Engineering, The Islamia University of Bahawalpur, Bahawalpur, 63100 Pakistan; 2 Department of Biomedical Engineering, Riphah College of Science and Technology, Riphah International University, Islamabad, 46000 Pakistan; 3 Institute of Cardiology Multan, Multan, Pakistan; 4 Department of Computer Science and Engineering, Mälardalens University, Box 883, 721 23 Västerås, Sweden

**Keywords:** Cardiovascular diseases (CVDs) MIMIC-IV-ECG dataset, Cardiovascular ECG data recovery and classification (CEDRC), Electrocardiogram (ECG), Machine learning (ML), Deep learning (DL), Waveform database (WFDB)

## Abstract

Cardiovascular diseases (CVDs) are a significant and widespread cause of death in the world, continuing to increase mortality rates. Therefore, timely identification and diagnosis are essential for a patient’s optimized recovery and longevity. In this regard, ECG is an effective tool for detecting anomalous heart conditions. However, interfering factors like noise, transient changes, and missing data could affect the accurate diagnosis of CVDs. The Cardiovascular ECG Data Recovery and Classification Network (CEDRC-Net) sorts and collates noisy data while also recovering missing data caused by equipment malfunction or human error, using a multistage machine-learning and deep-learning model. Moreover, CEDRC-Net is incorporated into the Transformer-based Convolutional Denoising Autoencoder (TCDAE) model to methodically mitigate noise, subsequently the Variational Autoencoder (VAE) or Temporal Fusion Transformer (TFT) are systematically employed as an alternative to accurately reconstruct and forecast ECG signals. Following this, the system classified heart diseases, including atrial fibrillation, sinus bradycardia, and tachycardia, using several machine-learning algorithms, based on data from a dataset comprising 2426 patients.TFT showcased better performance than VAE in ECG signal reconstruction, achieving up to 98% accuracy with Gradient Boosting, compared to 96% by VAE. Furthermore, in downstream classification, signals enhanced by TFT led to superior model results, with SVM and XGBoost both reaching 98.4% accuracy and F1-scores. The TFT achieved substantially lower reconstruction errors (MAE = 0.015, MSE = 0.00045, RMSE = 0.0132) compared to the VAE (MAE = 0.075, MSE = 0.011, RMSE = 0.107). These results highlight TFT’s strong denoising capability for improving ECG diagnostic accuracy, while the suggested system ensures reliable measurements under noisy and non-ideal conditions. It is highly conducive for early and accurate diagnosis, better clinical decisions, and decreased patient load on the cardiologists. The research is performed on the MIMIC-IV-ECG 12-lead real-time dataset.

## Introduction

**Fig. 1:**
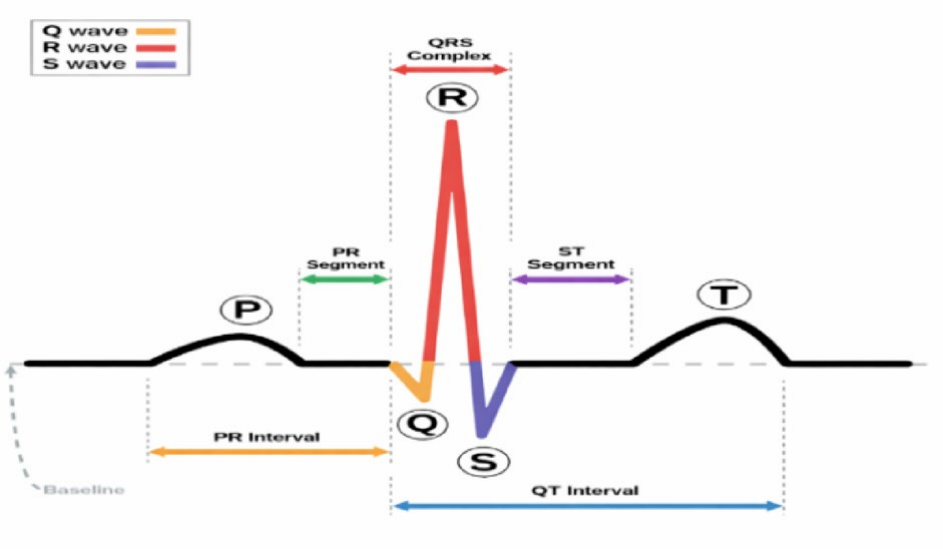
Normal ECG waves.

Electrocardiography (ECG) is a fundamental diagnostic approach in the identification and diagnosis of heart diseases. The 12-lead ECG is also called a gold standard diagnostic method that captures the electrical activity of the heart^1^. Moreover, the twelve electrodes provide a complete evaluation of the heart function and detect anomalies. It measures the heart’s electrical activity by positioning electrodes based on a set of predetermined guidelines^2,3^. These signals are classified into bipolar leads (I, II, III), augmented unipolar leads (aVR, aVL, aVF), and precordial leads (V1 to V6), based on the voltage difference between electrodes. At the same time, bipolar leads and augmented unipolar leads record the cardiac activity on the frontal plane, while precordial leads record the cardiac activity in the horizontal plane through six electrodes around the heart^4^. In this regard, Fig. 1 shows a standard electrocardiogram (ECG) waveform P wave, QRS complex, T wave, PR interval, ST segment, and QT interval illustrating different phases of cardiac electrical activity^5^.

The ECG is always used in diagnosis, prognosis and prevention of cardiovascular disease, nonetheless, obstacles persist, including a growing unmet need for proficient cardiologists who can reliably read ECGs, technical anomalies, such as poor contacts between electrodes due to patient movement, obesity, muscular contraction artefacts and equipment malfunctions, such distortions can lead to misdiagnosis or a delay in treatment. To overcome this issue researchers are applying multiple ECG reconstruction and denoising techniques for accurate diagnosis^6–8^.

Traditional ECG reconstruction and denoising algorithms, which are based on signal-processing principles, such as linear filtering, adaptive filtering and transform-based approaches, play a vital role in maintaining optimal signal integrity. The purpose of these methods is to remove noise in ECG signals, and enhance the quality of the data during analysis^9–14^. Even though the desired performance is not always attained by conventional signal processing methods, especially where non-stationary, complex noises or low-amplitude signals are involved^15^. The trend among researchers is changing to overcome the weaknesses of the conventional methods. The AI models are trained with large data sets to detect complex and subtle patterns and thus, offer more adaptive, flexible and specific solutions to denoising, reconstruction, and classification^16^.

To address these limitations, the recent studies have shifted to the focus on data-driven and AI-based approaches. Specifically DL models have produced substantial improvements in Signal-To-Noise Ratio (SNR) and Root Mean Square Error (RMSE) and retained the key clinical characteristics of P-waves, QRS complexes and T-waves hence making them clinically relevant and of great benefit^17–21^. Deep learning-based models such as GANs, and hybrid network techniques offer end-to-end learning and are considered for robust on several ECG benchmarks^22^.

In particular, CNNs, especially U-Net models, have been trained to complete missing segments of ECGs by taking advantage of the patterns obtained by analyzing known signal records and features of the waveforms^23^. Similarly GANs learn the distribution of natural ECG patterns, which allows them to be used to produce realistic replacements of absent data^24^. Likewise, VAEs have shown promise in reconstructing missing signals by modeling latent distributions^25^.VAEs provide a probabilistic generative model to impute missing ECG data^26^. Meanwhile, Transformer-based models utilize self-attention mechanisms to learn long-range dependencies in ECG signals^27^. Recent advancements, such as MuRe-LAT integrates convolutional feature extraction, lead-attention, and dual-scale transformer encoders to capture both local ECG morphology and long-term temporal dependencies for arrhythmia classification^28^. Likewise, HCTG-Net hybrid CNN transformer combines waveform morphology and temporal dynamics by combining residual convolutional feature maps with transformer-based gated fusion modules^29^. Other transformer-based ECG pipelines make use of attention-focused feature learning and optimized preprocessing pipelines to allow effective multi-class arrhythmia detection, and automated cardiac diagnostics^30^. Alongside, TFTs use gating mechanisms and multi-horizon attention to generate accurate forecasting of the missing data segments^31^.

The reviewed studies mainly focus on the developments in automated ECG-based detection and classification of cardiovascular diseases, including arrhythmia, heart failure and atrial fibrillation, by the use of the state-of-the-art deep learning architectures. Despite these advancements, there are still serious limitations, such as low interpretability, imbalanced classes, and low cross-dataset generalizability. Past literature also shows poor multimodal contextual information integration^32^.Similarly excessive use of complicated preprocessing and poor long-range temporal modeling^33^, poor inter-lead structural-dependency^34^, and significant computational cost that makes it difficult to use in practice^35^.

The proposed CEDRC-Net is a multi-stage framework designed for ECG denoising, missing data reconstruction, and cardiac disease classification, trained on the MIMIC-IV-ECG dataset comprising over 800,000 ECG recordings^36^. The model analyzes a variety of missing-data conditions and compares reconstructed signals to the original ones to assess predictive performance. Another goal is to increase diagnostic accuracy, decrease workload in clinicians and provide early intervention in cardiovascular disease (CVD).

## Literature review

The unique technological development has triggered tremendous changes in the field of medical science. The current trends in deep learning have significantly enhanced the reconstruction of electrocardiogram (ECG) signals in various situations. Here, Guo et al. have shown that a WNet -BiLSTM model is capable of effectively reconstructing missing ECG segments from (PPG) data with a Pearson correlation of 0.851 and root-mean-square error (RMSE) of 0.075 when given 3 s gaps between ECG segments^20^. Similarly, in another significant study, a U-Net architecture on the PTB-XL dataset has been able to recreate missing ECG leads with an R^2^ of 0.94 and an RMSE of 0.037 mV, thus demonstrating the fine-grained signal reconstruction ability of the model^37^.

Moreover, a hybrid LSTM-UNet model demonstrated an R^2^ of 94.37% when tested on the PTBDB dataset, thus further supporting the effectiveness of combining sequential and convolutional architectures to recover lead. In order to overcome the poor quality of data or missing data, a hybrid Bi-LSTM and CNN model was trained using 13,862 recordings with an RMSE of 0.037 u V and a cosine similarity of 0.991, indicating a high level of fidelity of waveforms^1^. The study by U. Lomoio et al. proposed a solution to improve resolution in deteriorated signals, a Denoising Convolutional Autoencoder (DCAE-SR) achieved a signal-to-noise ratio of 12.20 dB, a mean squared error of 0.0044, and a root mean squared error of 4.86%, demonstrating robust performance in enhancing signals under noisy conditions^38^. Furthermore, the research presented by M. Dias et al. introduced using recurrent networks, a biGRU-based denoising model trained on PTB-XL data with synthetic noise from the MIT-BIH Noise Stress Test Database attained RMSEs between 0.023 and 0.041, outperforming standard open-source benchmarks^39^. Additionally, to corroborate the previous assertions, another study by A. Ezzat et al. developed an attention-based CNN-BiLSTM model that used wavelet scattering transform features to reconstruct ECG from PPG signals, achieving an RMSE of 0.031 and demonstrating improved signal fidelity compared to previous frameworks^40^.

Furthermore, C. J. Harvey et al. opined in their research that VAEs have become powerful tools for ECG signal recovery and representation learning. They compress ECG signals into latent spaces while reconstructing the inputs, thereby aiding denoising and preserving clinical features^41^. Moreover, the study by A. Habib et al. examined the Advanced VAE architectures improving the long-recording handling and feature interpretability. A folded CNN-VAE processes 30-s ECGs in segments, enhancing reconstruction accuracy^42^. Similarly, a novel study by A. Nazabal et al. proposes HI-VAE, a Variational Autoencoder framework tailored to handle heterogeneous and incomplete data using a hierarchical generative model and Gaussian mixture priors^43^. The study by L. Chen et al. VAEs have also been applied to ECG delineation: one variational encoder-decoder network achieved state-of-the-art accuracy in detecting QRS onsets and T-wave peaks in varying morphologies^44^.

In the recent past, Transformer models, particularly TFT enhances model performance and interpretability by filtering irrelevant features, focusing on key data points through attention mechanisms, and offering insights into temporal patterns, significant for understanding medical predictions^31^. The TFT is also designed to handle time-series data like ECG signals, which exhibit both short-term and long-term dependencies, and by combining recurrent layers and self-attention mechanisms, TFT captures local and global temporal relationships in ECG signals^21^.

The research conducted by D. Rathore et al. demonstrated that Machine Learning (ML) models are an effective approach for ECG classification^45^. In the study by Z. S. Munmun et al. examined the classification of heart-diseases and used a standard Random Forest model, with a high level of generalization in cross-validation, a macro-F1-score of 95%, a precision of 96% and a recall of 97%. These findings indicate a high level of performance in detecting diseases and at the same time decrease false positives and false negatives^46^. The study by Y. Rimal et al. designed a genetic-algorithm-optimized Random Forest (GA-RF) model to predict coronary artery disease with an accuracy of 92 per cent by using the hyper parameter optimization and advanced feature selection to fit the model to particular data sets like the UCI and Cleveland data sets^47^. According to the researchers, the growing use of machine-learning algorithms to classify the cardiovascular disease (CVD) leads to different levels of accuracy. Like, a stacked ensemble model (ExtraTrees, Random Forest, XGBoost) achieved a classification accuracy of 92.34%, indicating the potential of ensemble approaches, although further optimization is required to reach higher accuracy levels^48^. Similarly, The study by J. Miah et al. presents a comparative analysis of six ML classifiers for myocardial infarction prediction, with XGBoost achieving the highest accuracy of 92.72%, followed closely by LightGBM at 90.60%^49^. Moreover, The study by B. Chulde-Fernández et al. a machine learning framework is developed to classify heart failure, with the Multi-Layer Perceptron (MLP) model reaching an accuracy of 94%^50^.

On the other hand, the article by W. Caesarendra et al. delves into the analysis of Deep Learning models with significant potential in the classification of ECG signals, especially in the detection of arrhythmia and patient-specific diagnosis. A CNN trained on PhysioNet database with 100 epochs was 95% accurate in classifying ECGs among four classes^51^. M. Kolhar et al. delineate the Convolutional Neural Networks (CNNs) that have shown strong performance in electrocardiogram (ECG) classification tasks due to their ability to extract complex features from signal patterns^52^. On the same note, it can be found that a multiclass CNN classifier achieved about 96 percent accuracy and a inference time of less than a second, thus making it more useful in real-time monitoring, by the investigation of M. D. M. Qureshi et al.^53^. Moreover, P.N.Malleswari et al. introduce an ensemble model that uses a 1D CNN on ECG signals and a 2D CNN on ECG images, using transfer learning to achieve 94% and 93% accuracy on five cardiac classes each, respectively, thus confirming the advantage of multimodal inputs^54^. Additionally, the research by F. Khan et al. presents a noise-resilient 1D CNN trained on augmented MIT-BIH data which attained 94% accuracy on four arrhythmic classes and is resilient in noisy conditions^55^. B. Manjesh in one of his work, demonstrates the effectiveness of an RNN model which reached an accuracy/AUC of about 96 to classify arrhythmias based on ECG and other biosignals and the sensitivity of a cardiologist to some arrhythmias^56^. Likewise, Y. Dong et al. build on an arrhythmia classifier using a vision transformer with deformable attention that showed a high accuracy and F1 -scores of multiple arrhythmia types on the 12 -lead PTB -XL dataset^57^.

Some studies show that intelligent feature engineering, memory-enhanced metaheuristic optimization, and advanced deep learning can achieve near-clinical accuracy in ECG-based arrhythmia detection.Kaya et al. designed a bundle branch block detection system using 32 statistical–temporal features from 200-sample MIT-BIH heartbeats, with Forward/Backward Selection and Genetic Algorithms for feature optimization. Under tenfold cross-validation, the Backward Elimination–KNN model achieved 99.82% accuracy, highlighting the effectiveness of structured feature selection^58^. Another study Kıymaç et al. proposed a memory-enhanced Artificial Hummingbird Algorithm to optimize CNN hyperparameters for MIT-BIH arrhythmia classification. By minimizing redundant evaluations and incorporating model complexity into the fitness function, the method reached 98.87% accuracy, outperforming multiple metaheuristics^59^.Similarly, Lamba et al. introduced the FADLEC framework a deep learning approach for five-class arrhythmia classification (N, S, V, F, Q) using MIT-BIH data. After wavelet denoising, Z-score normalization, SWT-HT (Stationary Wavelet Transform—Hilbert Transform) feature extraction, and SMOTE (Synthetic Minority Over-sampling Technique)-based imbalance correction, Bi-LSTM and FCN models optimized via Ant Colony Optimization achieved accuracies of 98.9% and 99.1%, respectively, surpassing existing methods^60^.

Recent studies in the electrocardiography signal processing field show that there is a shift to a paradigm where isolated and task-specific models are replaced by a framework that considers signal representations, which deal with denoising, reconstruction and classification in clinically based pipelines. Denoising is also being positioned as latent-representation disentangling as opposed to traditional filtering; morphological features being maintained in the presence of mixed artefacts is still a key challenge. The current focus of reconstruction studies on downstream diagnostic validity along with the similarity of waveforms has indicated the shift toward the idea of signal fidelity being replaced by a wider perspective on clinical relevance. Even though convolutional neural networks, long-short-term memory networks, hybrid networks, meta-heuristic optimization algorithms, variational auto-encoders and transformer-based architectures demonstrate significant performance improvements, recent results suggest that accuracy is not enough. The tests of robustness, sensitivity to the imbalance of the classes, and cross-dataset validation are critical and the alleged superiority of transformers depends, in fact, on the concrete task situation greatly.

In this context, the proposed modular, stage-wise framework advances the field by integrating representation-based denoising, generative (VAE) or attention-driven (TFT) reconstruction, and interpretable, computationally efficient classifiers within a unified pipeline, enabling morphology preservation, controlled ablation, improved robustness to noise and missing leads, enhanced generalization, and practical clinical deployment.

## Methodology framework (CEDRC-Net)

**Fig. 2:**
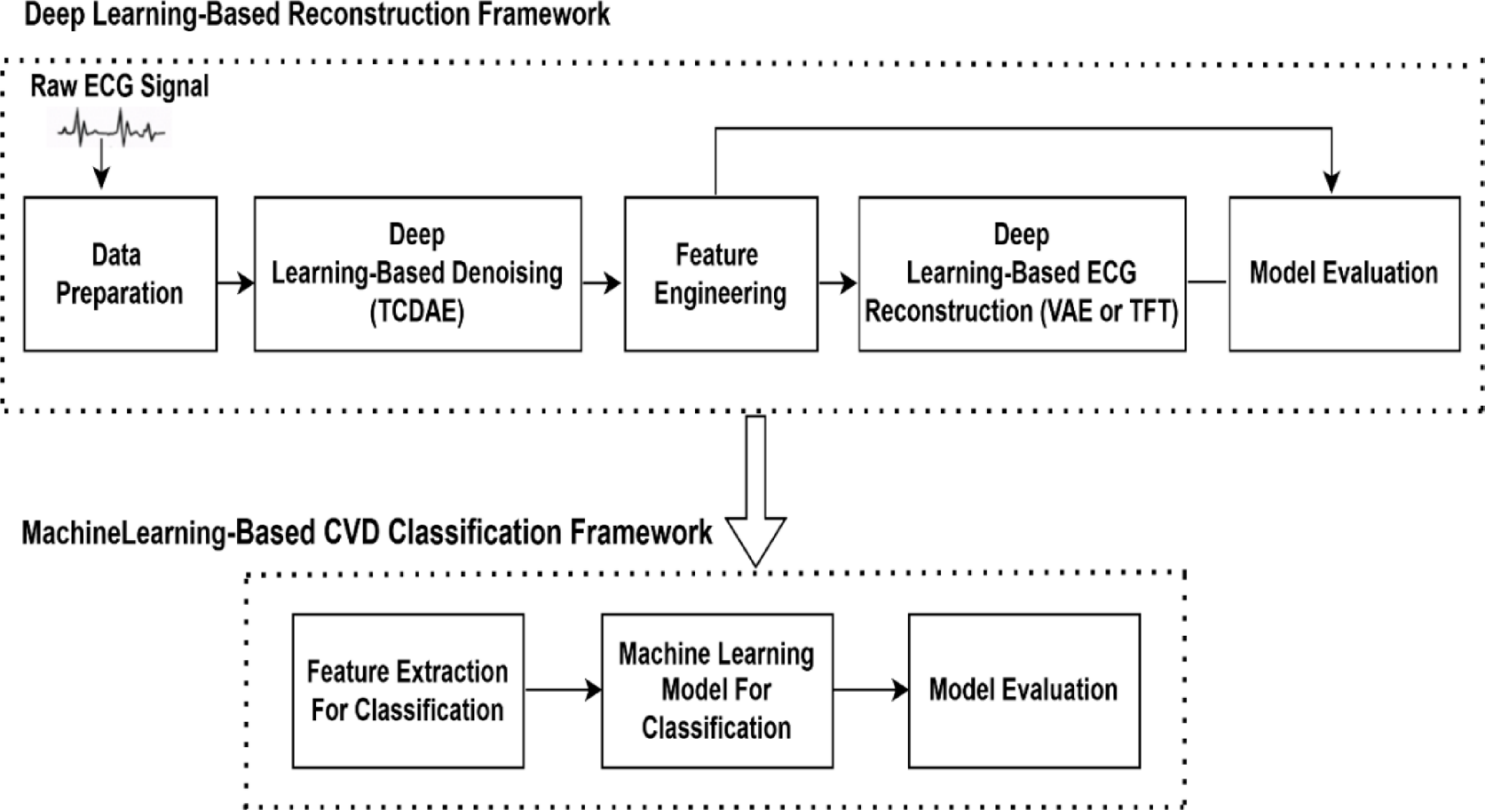
CEDRC-Net framework diagram.

The main aim of the current research is the effective diagnosis and proper interventions in case of cardiovascular disease. The Fig. 2 illustrates a two-stage ECG analysis pipeline consisting of a deep learning–based reconstruction framework followed by a machine learning–based CVD classification framework. The first step is the processing of raw ECG data to structured data, which involves segmentation, normalization, and partitioning of the datasets, thus providing consistency and preventing data leakage. The resulting preprocessed signals are then sent to a Temporal Convolutional Denoising Autoencoder (TCDAE). This auto-Encoder reduces noise and artefacts and maintains necessary morphological features like P-waves, QRS complexes and T-waves. Discriminative statistical, temporal and morphological features are designed out of the denoised signals. Then, a rich generative or attention-based reconstruction model, such as a Variational Autoencoder (VAE) or a Temporal Fusion Transformer (TFT), is trained on small latent representations to generate fine ECG waveforms. The result of this process is a higher level of signal fidelity and enables contextual temporal modelling. The performance of reconstruction is measured using quantitative metrics and the feedback obtained optimizes the previous phases. The second stage is the reconstruction of the ECG which serves as an input to the classification pipeline. In this case, more features are obtained and used in a machine learning classification model that is aimed at identifying cardiac abnormalities. Performance of the model is eventually evaluated. This twin-model framework enhances the quality of the signal and the ability of diagnostic results.

### Dataset, file structure and reading tool information

Data is one of the most essential elements in the creation of machine learning and deep learning solutions. The data required for models should include raw ECG, patient demographic, clinical data, and possibly labels that characterize some pathological condition or health condition. The MIMIC-IV-ECG data is a large-scale collection containing about 800,000 diagnostic ECG recordings of almost 160,000 individual patients. The recording of each ECG is done with 12-lead configuration, 10 s, and a sampling frequency of 500 Hz^36^. This subgroup includes all ECGs of patients who are simultaneously present in the MIMIC-IV Clinical Database. Whenever an ECG is accompanied by an interpretive report prepared by a cardiologist, the waveforms are overlaid on a grid which defines the time ranges and amplitude of the voltages, thus making it easier to morphologically evaluate the ECG tracings.

. A 12-lead ECG with standard leads (I, II, III, aVR, aVL, aVF) and the precordial leads V1 through V6 plots time (in seconds) on the x-axis, with voltage (in millivolts, mV) on the y-axis. Each waveform exhibits the characteristic peaks and troughs typical of ECG signals, including P waves, QRS complexes, and T waves.

ECG data in the MIMIC-IV dataset is stored in a header file containing key metadata such as record name, number of signals, sampling frequency, sample count, and optional fields. Each signal also has a per-signal header specifying the signal file name, format, gain, baseline, units, initial value, checksum, and block size. The Waveform Database (WFDB) is used to read and write records and annotations from raw ECG files and retrieve available records from PhysioNet. To avoid data leakage, the MIMIC-IV dataset is split at the patient level, ensuring that records from patients with similar diseases are not shared between training, validations, and testing sets. The dataset is divided into 70% training, 10% validation, and 20% testing patients.

### Denoising process

**Fig. 3:**
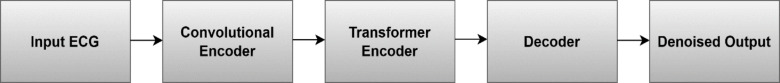
Model architecture of TCDAE.

TCDAE is suggested to reduce random mixed noise in ECG signals by training a mapping between noisy and clean segments of the signals^61^. Figure 3 shows the TCDAE model that performs the denoising of the ECG signal. The architecture is an encoder transformer decoder framework, where the encoder uses three gated convolutional layers to obtain local ECG morphological characteristics and suppress noise. The features extracted are then fed through a Transformer encoder with multi-head self-attention and positional encoding to encode long-term temporal relationships in the ECG sequence. A decoder is used to reconstruct the denoised signal with transposed convolutional layers with skip connections, and the waveform details are preserved. In training, a combined loss function of frequency-weighted Huber loss and cosine similarity loss is used to reduce reconstruction loss without compromising both the temporal and spectral properties of the ECG signal to achieve the ability to remove mixed noise with minimal signal distortion.

**Fig. 4:**
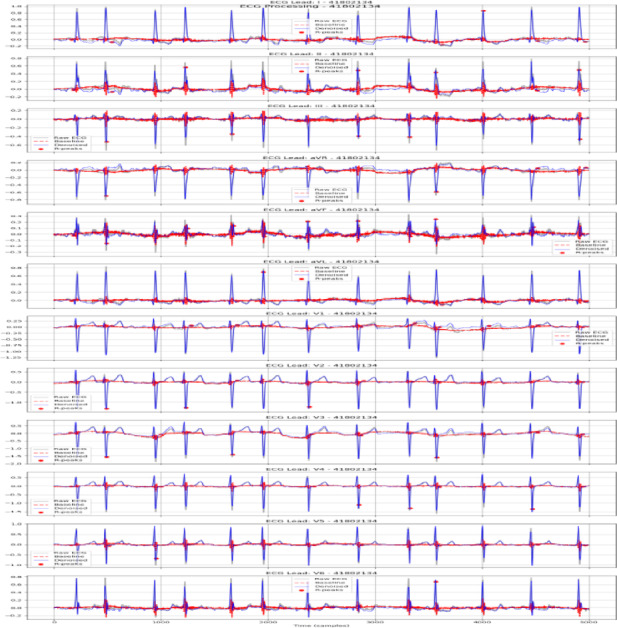
Raw and denoised ECG.

**Fig. 5:**
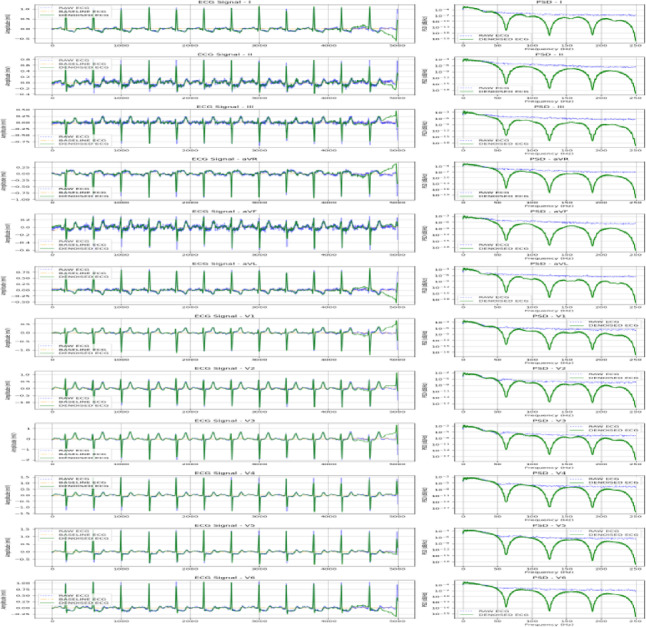
Validation denoised data.

Figures 4 and 5 demonstrate that the TCDAE model effectively denoises ECG signals while preserving the waveform morphology. The power spectral density (PSD) analysis confirms significant noise reduction in the frequency domain. Further improvement can be achieved by incorporating frequency-weighted loss to better balance noise suppression and preservation of important ECG features.

In Table 1, conduct cross-validation by comparing cleaned data with the original files to ensure no critical loss, and complement it with visualizations to confirm data integrity and indicate substantial signal similarity. Mean Absolute Error (MAE) values are low across leads, confirming effective noise reduction with minimal signal distortion.

**Table 1:** Validation of denoised data.

Lead	ECG	Denoised ECG	Energy Diff (%)	Pearson	MAE
I	4723.84	4251.46	10	0.92	0.1
II	4640.7	4176.63	10	0.91	0.105
III	4653.65	4188.83	10	0.9	0.11
aVR	4657.97	4192.17	10	0.93	0.095
aVF	4641.02	4176.92	10	0.905	0.1075
aVL	4695.48	4225.93	10	0.915	0.1025
V1	4587.72	4128.95	10	0.94	0.09
V2	4621.47	4159.32	10	0.925	0.0975
V3	4618.01	4156.21	10	0.9	0.11
V4	4640.49	4176.44	10	0.895	0.1125
V5	4663.74	4197.37	10	0.91	0.105
V6	4848.91	4364.02	10	0.89	0.115

### Simulating missing ECG segments

To simulate missing segments in the ECG test dataset, we use a pseudorandom number generator (typically the Mersenne Twister) to select indices from the dataset without replacement randomly. This ensures that segments are removed automatically rather than manually. To avoid repeating the same index, we apply sampling without replacement using techniques like the Fisher-Yates shuffle.”

### Feature engineering

Extract relevant metrics from the raw ECG data, such as heart rate and QRS complex, P-Wave and T-Wave, which are essential for diagnosing cardiac conditions.

## Preparation for multi-stage model

### Variational autoencoder

**Fig. 6:**
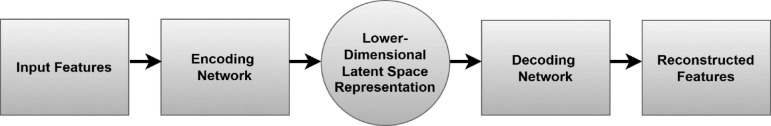
Model architecture of VAE.

The **VAE** component reconstructs missing segments in 12-lead ECG signals by learning a compressed latent representation and generating complete waveforms through probabilistic decoding. Figure 6 depicts the VAE architecture for reconstructing missing ECG signals using a latent space representation.

#### Problem formulation

Let $\text{X }\in {\mathrm{R}}^{ T\times L}$ denote a 12-leads ECG recording, where $\text{X is the input data},$ T is the number of Time and samples and L is the number of leads. We defined a binary mask $\text{M }\in {\{0, 1\}}^{\text{T }\times \mathrm{L}}$ indicating observed ${(M}_{t,l}=1)$ and the missing ${(M}_{t,l}=0)$ values. The observed data is denoted as ${\mathrm{X}}_{\mathrm{obs}}=\mathrm{M}\odot \mathrm{X}$. The symbol $\odot$ means element-wise multiplication.

#### Generative model

The VAE models the joint distribution of ECG data **X** and latent variables $\mathrm{z}\in {\mathrm{R}}^{ K}$, where z represents a latent variable and $K$ represents the dimension of the latent space. ${P}_{\theta }\left(X, z\right)={ p}_{\theta } (X | z) p(z)$, Where ${p}_{\theta } (X | z)$ Is probability that the decoder generates X from z and $p(z)$ is the prior distribution over the latent space, typically modeled as an isotropic Gaussian distribution, $p(z)=N\left(z; 0, {I}_{K}\right)$ where $0\in {R}^{k}$ and ${I}_{K}\in {R}^{k*K}$ is the identity matrix and *N* denotes the Normal distribution, also called the Gaussian distribution.

*Decoder Likelihood* The decoder reconstructs the complete ECG signal from the latent representation. We assume a Gaussian likelihood with diagonal covariance is mentioned in Eq. (1).

1 $$p_{\theta } (X | z) = N (X; \mu_{\theta } \left( z \right), diag\left( {\sigma_{\theta }^{2} \left( z \right)} \right)$$

where ${\mu }_{\theta }$ is the decoder neural network outputting the reconstructed ECG mean,${\sigma }_{\theta }$ shows the reconstruction uncertainty and $\theta$ denotes the decoder parameter (weight and biases).

#### Inference model

*Variational Posterior* The encoder approximates the intractable true posterior ${P}_{\theta }(z | {X}_{obs })$ with a Gaussian as mentioned in Eq. (2).

2 $${q}_{\phi }(z | {X}_{obs})=N (z;{\mu }_{\phi }({X}_{obs}), diag({\sigma }_{\phi }^{2}\left({X}_{obs}\right)))$$

where ${q}_{\phi }(z | {X}_{obs})$ is posterior distribution produced by the encoder network,${\mu }_{\phi }\left({X}_{obs}\right)$ denotes the mean predicted by the encoder and ${\sigma }_{\phi }^{2}\left({X}_{obs}\right)$ variance predicted by the encoder.

Equation (3) is representing the *reparameterization trick* used in VAE to sample the latent variable z.${\mu }_{\phi }\left({X}_{obs}\right)$ mean produced by the encoder network,${\sigma }_{\phi }\left({X}_{obs}\right)$ standard deviation produced by the encoder and ϵ denotes the random noise sampled from a standard normal distribution.

3 $$z = {\mu }_{\phi }\left({X}_{obs}\right)+{\sigma }_{\phi }\left({X}_{obs}\right)\odot \epsilon , \epsilon \sim N (0,{ I}_{K})$$

#### ECG reconstruction

Equation (4) providing the reconstructed ECG signal is obtained as the mean of the decoder distribution, the information of decoder ${\mu }_{\theta }$ maps the latent representation z back into the reconstructed ECG signal $\widehat{X}$ representing the model’s estimate of the cleaned or enhanced ECG.

4 $$\hat{X} = \mu_{\theta } \left( z \right)\,{\mathrm{Where}}\,z\sim q_{\emptyset } (z|X_{obs} )$$

#### Loss function

The VAE’s loss function includes both the reconstruction loss and the KL divergence between the posterior and prior distributions. Equation (5) defines the total VAE loss function, combining reconstruction loss and KL divergence.

5 $${L}_{VAE} ={L}_{recon }+ \beta .{D}_{KL}({q}_{\phi }(z|{X}_{obs})\parallel p(z))$$

where *β* is a hyperparameter controlling the trade-off between reconstruction accuracy and latent regularization (*β* = *1* for standard VAE).

### Temporal fusion transformer

**Fig. 7:**
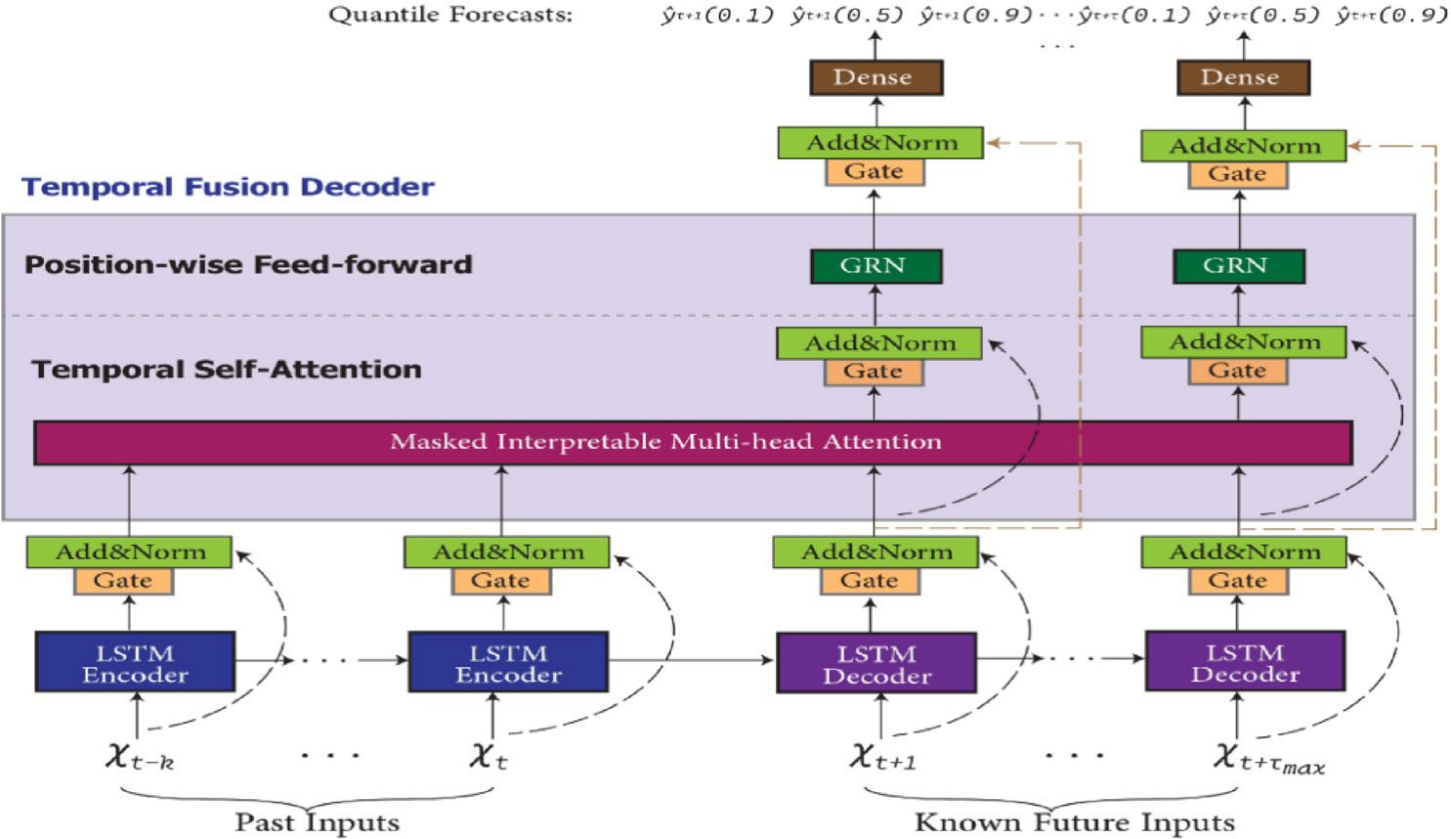
Model architecture of TFT.

TFT can be used for multivariate time-series forecasting and handles missing data during prediction. It combines LSTM encoders and decoders to process past and future inputs, with multi-head attention to capture long-term dependencies. The Temporal Fusion Decoder integrates gated residual networks (GRNs) and attention mechanisms to generate accurate, interpretable quantile forecasts of the missing data segments^31^. Figure 7 illustrates the TFT architecture, moreover TFT components can be used for missing ECG recovery as well.

#### Input representation

Let ${x}_{t}\in {R}^{L}$ denote the ECG amplitude vector at time step t for leads, a sequence of length is used for *Historical Context*: ${\left\{{X}_{t}\right\}}_{t=1}^{{T}_{obs}}$ where ${X}_{t}= \left\{{x}_{t}^{1} , {x}_{t}^{2},\dots . . . , {x}_{t}^{n} \right\}, {T}_{obs}$ is the observed window length and *Prediction horizon*: $\tau \in \left\{1,\dots \dots {\tau }_{max}\right\}$ where $\tau \text{is time step}$ $, {\tau }_{max}$ is the number of missing time steps to reconstruct. A *mask vector* is also define which is ${m}_{t}\in {\left\{\mathrm{0,1}\right\}}^{L}$ indicate missing values at time *t*.

#### Variable selection networks

For each lead $l\in \left\{1,\dots \dots .,L\right\}$ at time *t.* The variable selection network computes importance weights ${v}_{t}=softmax({GRN}_{v}\left({E}_{t,}{c}_{s}\right))$, where ${E}_{t}={\left[{x}_{t}^{1}\dots ..{x}_{t}^{L} \right]}^{T }\in {R}^{L*{d}_{model}}$ contains the embedded lead features and $C_{S}$ is the static context vector from lead-invariant features. The selection features are shown in Eq. (6), where ${x}_{t}$ is final selected and weighted feature,${v}_{t}^{l}$ is weight for feature and ${x}_{t}^{l}$ is the input feature and ${GRN}_{sel}$ is used to transforms each feature to extract useful information.

6 $$\widetilde{{x}_{t}}=\sum_{l=1}^{L}{v}_{t}^{l }.{GRN}_{sel}\left({x}_{t}^{l}\right)$$

#### Interpretable multi-head attention

The TFT employs multi-head self-attention to model temporal dependencies across ECG time steps. For head $h\in \left\{1,\dots \dots ,{m}_{H}\right\}$. Equation (7) computes attention weights between queries and keys, then uses them to aggregate the value vectors, enabling the model to capture long-range dependencies in sequential data. Q, K, and V denote the Query, Key, and Value matrices,${W}_{Q}^{h}$**,**${W}_{k}^{h},$ and ${W}_{v}^{h}$ are learnable projection matrices that transform the input features into query, key, and value representations, while $Q{W}_{Q}^{h}{(K{W}_{k}^{h})}^{t}$ computes similarity scores between queries and keys to determine the attention strength between sequence. $\sqrt{{d}_{attn}} \mathrm{is}$ Scales attention scores for stability. $V{W}_{v}^{h}$ Projects and aggregates value vectors using these weights, and ${Attention}_{h}\left(Q,K,V\right)$ produces the final context-aware representation for attention head h.

7 $${Attention}_{h}\left(Q,K,V\right)=softmax\left[Q{W}_{Q}^{h}{(K{W}_{k}^{h})}^{t}/\sqrt{{d}_{attn}}\right]V{W}_{v}^{h}$$

The interpretable multi-head attention averages across heads is given in Eq. (8)

8 $$\widetilde{H}=\frac{1}{{m}_{H}}\sum_{h=1}^{mH}{Attention}_{h}\left(Q,K,V\right)$$

#### Static enrichment

Temporal features are enriched with static lead information as mentioned in Eq. (9) Where ${\widetilde{h}}_{t}$ the output of the interpretable attention is at time ${c}_{e}$ is the static enrichment context.

9 $$\theta \left(t\right)={GRN}_{enrich}({\widetilde{h}}_{t},{c}_{e})$$

#### Quantile forecasting for ECG reconstruction

Each lead and quantile $q\in Q$ the model predicts. ${\widehat{x}}_{t+\tau ,l}^{q}={f}_{q,l}(\theta \left(t\right),{c}_{h}^{\tau }$ Where ${f}_{q,l}$ denotes the linear projection for quantile q and lead $l$, and ${c}_{h}^{\tau }$ ​ is the horizon-specific context vector produced by the attention mechanism. The final reconstruction is typically obtained using the median.

#### Loss function

The TFT is trained using the quantile loss summed over all leads and prediction horizons:

10 $${L}_{TFT}= {\sum }_{t={T}_{obs}+1}^{{T}_{obs}+{\tau }_{max}} {\sum }_{l=1}^{L} {\sum }_{q\epsilon Q} {p}_{q}\left({x}_{t,l}-{\widehat{x}}_{t,l}^{q}\right).{\mathrm{I}}_{[t,l,obs]}$$

The loss ${L}_{TFT}$ is computed by summing the quantile loss ${p}_{q}$ between the true value ${x}_{t,l}$ and the predicted quantile value ${\widehat{x}}_{t,l}^{q}$ for each time step t, lead $l$**,** and quantile $q$**.** The indicator term ${\mathrm{I}}_{[t,l,obs]}$] ensures that the loss is calculated only for observed data points.

The TFT architecture is technically adapted for ECG reconstruction through.

- *Bidirectional encoding* to leverage both past and future cardiac cycles
- *Explicit segment masking* with learnable masking tokens for missing data representation
- *Lead-specific static covariates* encoding anatomical and physiological lead characteristics
- *Cardiac-cycle-aware positional encodings* that respect heart rate periodicity
- *Causal attention masking* preventing attention to missing segments during reconstruction
- *Quantile regression outputs* providing probabilistic reconstructions with uncertainty estimates

These modifications transform TFT from a forecasting model into an imputation model specifically designed for the temporal and spatial characteristics of 12-lead ECG signals, while preserving the interpretability and multi-horizon capabilities of the original architecture.

### Feature engineering for classification

After getting the recovered ECG from the VAE or TFT algorithm, Hand-engineered morphological and temporal ECG features are extracted from the reconstructed signals using a validated ECG delineation pipeline implemented in the WFDB toolbox with Pan–Tompkins QRS detection^62^. The necessary features used for classification are also summarized in Table 2^63^.

**Table 2:** Common ECG signal features and their relevance to ECG analysis.

Feature	Description	Relevance to ECG Analysis
Bandwidth	Indicates the frequency range (in Hz) of the ECG signal. This can vary depending on the device or configuration	Higher bandwidth can capture more details of the ECG signal, while lower bandwidth may result in loss of finer features
Filtering	Describes the type of filtering applied to the signal (e.g., low-pass, high-pass, or band-pass filters)	Filtering removes noise and artifacts, making the signal clearer for analysis and feature extraction
rr_interval	The time (in seconds) between successive R-peaks in the ECG signal	A key measure of heart rate variability (HRV), which provides insights into autonomic nervous system activity
p_onset	The time (in seconds) at which the P-wave begins	Indicates the start of atrial depolarization, crucial for assessing atrial activity and diagnosing atrial abnormalities
p_end	The time (in seconds) at which the P-wave ends	Marks the completion of atrial depolarization; abnormalities in duration can indicate atrial disorders
qrs_onset	The time (in seconds) at which the QRS complex begins	It represents the start of ventricular depolarization, essential for detecting ventricular conduction delays
qrs_end	The time (in seconds) at which the QRS complex ends	Marks the completion of ventricular depolarization; prolonged QRS duration can signal conduction system abnormalities
t_end	The time (in seconds) at which the T-wave ends	Indicates the end of ventricular repolarization; changes in this feature can be associated with electrolyte imbalances or ischemia
p_axis	The electrical axis of the P-wave, measured in degrees	Reflects the direction of atrial depolarization, which can help in diagnosing atrial enlargement or conduction issues
qrs_axis	The electrical axis of the QRS complex, measured in degrees	Shows the direction of ventricular depolarization; deviations can indicate conditions such as hypertrophy or infarction
t_axis	The electrical axis of the T-wave, measured in degrees	Represents the direction of ventricular repolarization, which may shift in conditions like ischemia or pericarditis

11 $$y = f (bandwidth, filtering, rr interval,{p}_{onset} , {p}_{end}, {qrs}_{onset},{qrs}_{end},{t}_{end}, {p}_{axis}, {qrs}_{axis},{t}_{axis} ; \Theta )$$

Here, Eq. (11) represents the output y as a function of various ECG morphological and temporal features, including intervals, onsets, ends, and electrical axes. It represents how these parameters collectively determine the ECG based model output under the parameter set $\Theta$. The output y represents the predicted class, identifying the type of heartbeat or cardiac condition, such as a regular rhythm or a specific arrhythmia, as specified in the Eq. (12).

12 $${\mathrm{y}} \in \left\{ {{\text{Class }}0,{\text{ Class 1}},{\text{ Class 2}}, \, . \, . \, .} \right\}$$

Moreover, the Eq. (13) is presenting that the classification model aims to minimize the error between predicted values y^​and true values y by optimizing the loss function L, such as cross-entropy loss.

13 $$L(y,\widehat{y} ) = - {\sum }_{i=1}^{N} {y}_{i} log({\widehat{y}}_{i})$$

where N is the total number of samples, ${y}_{i}$ is the actual label, and ${\widehat{y}}_{i}$ is the predicted probability for class III. This equation, combined with the feature set, defines the relationship between the features and the target variable in this classification task.

## Results

Cardiovascular ECG Data Recovery and Classification Network (CEDRC-Net) is a multi-stage model dedicated to reconstructing and classifying the ECG signal efficiently. It performs better in restoring lost ECG signals through advanced architectures in improving the accuracy of CVDs diagnosis.

### Variational autoencoder

**Fig. 8:**
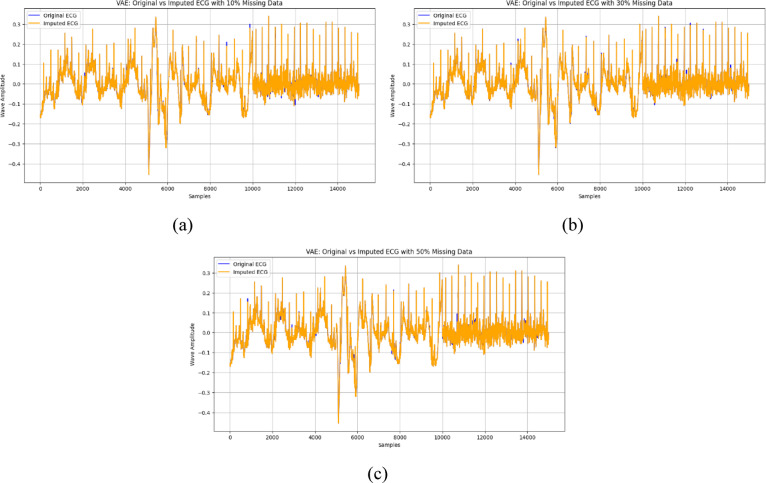
(**a**) 10%, (**b**) 30% and (**c**) 50% missing data prediction by VAE.

The performance of a VAE in reconstructing ECG signals with missing data is shown in Fig. 8. Each subplot compares the original ECG with the imputed ECG. The reconstruction quality drops slightly as the missing ratio increases; however, the model still captures waveform patterns, even with 50% data loss. The findings indicate that VAE is strong in imputing ECGs.

### Temporal fusion transformer

**Fig. 9:**
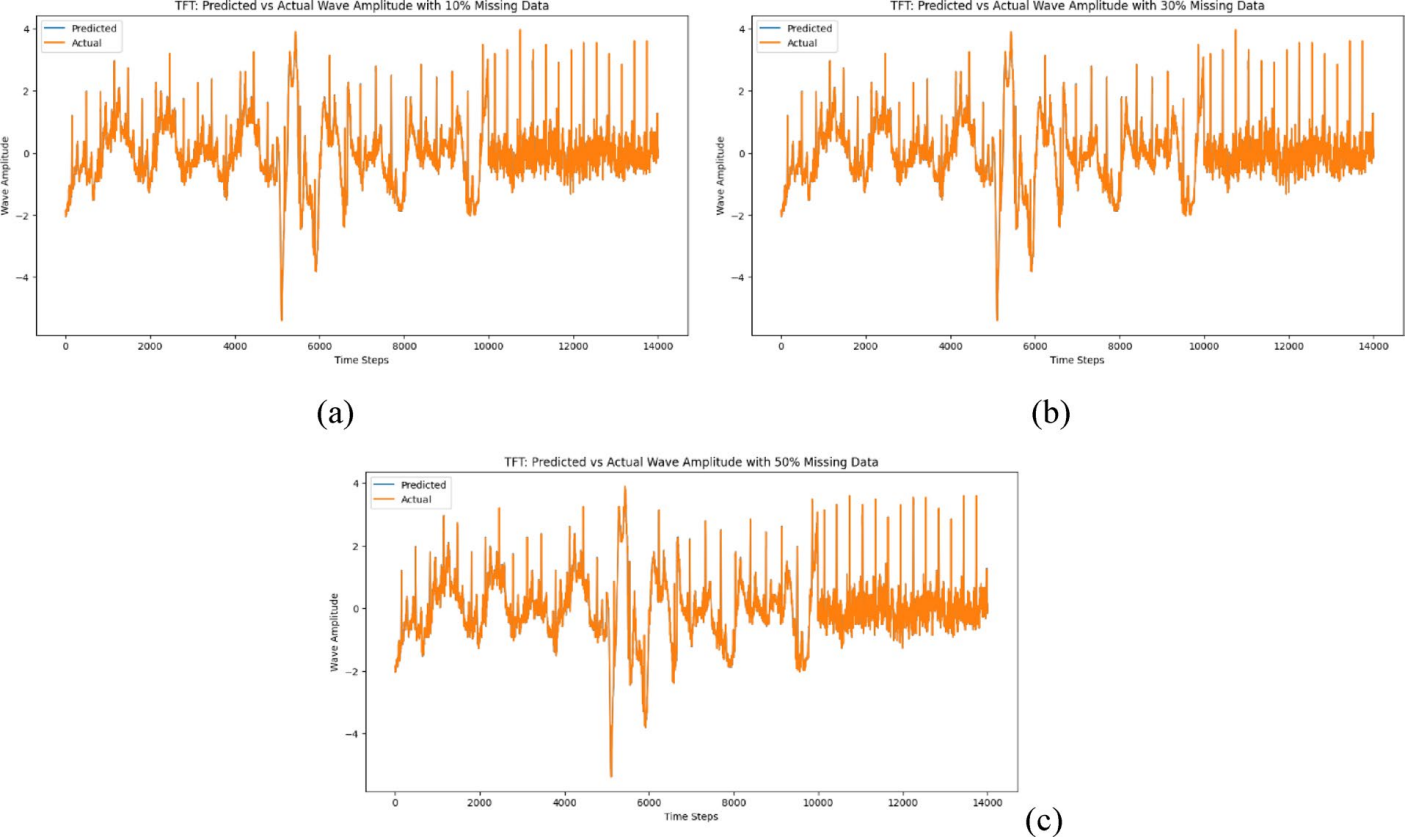
(**a**) 10%, (**b**) 30% and (**c**) 50% missing data prediction by TFT.

In the same manner, Fig. 9 also shows the results of the Temporal Fusion Transformer (TFT) to reconstruct electrocardiogram (ECG) signals with missing data. Each plot compares the predicted ECG to the actual signal over time. At 10% of the missing data, the prediction closely matches the actual signal. As missing data increases to 30% and 50%, the prediction deviates slightly, with more amplitude discrepancies. Despite this, the TFT retains the underlying waveform structure, demonstrating its robustness in handling incomplete ECG data and generating reliable reconstructions.

### Evaluation metrics of VAE and TFT

The performance of the VAE and TFT models is evaluated using the Mean Squared Error (MSE), Mean Absolute Error (MAE), Root mean square Error (RMSE) and Loss.

All ECG signals are normalized to the [0, 1] range prior to training and evaluation to ensure consistent scale across samples. As shown in Table 3, the TFT achieved substantially lower reconstruction errors (MAE = 0.015, MSE = 0.00045, RMSE = 0.0132) compared to the VAE (MAE = 0.075, MSE = 0.011, RMSE = 0.107). Furthermore, the normalized root mean square error (NRMSE) of 0.0167 indicates that the reconstruction error corresponds to approximately **1**.67% of the normalized ECG signal amplitude**,** demonstrating high reconstruction fidelity and improved clinical interpretability. This swift convergence indicates that the model effectively captures underlying data patterns early in the training process and achieves stable optimization during training.

**Table 3:**
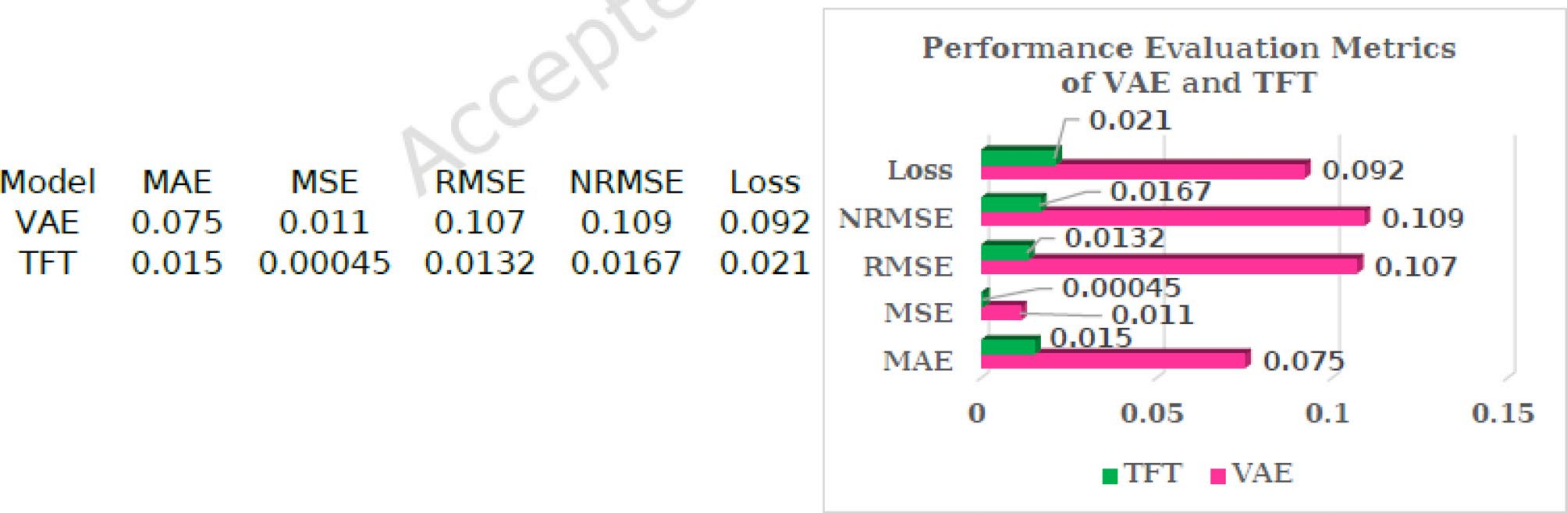
Performance comparison of VAE and TFT.

### Model evaluation for classification

This study investigates the performance of two prominent deep-learning models, VAE and the TFT in the reconstruction of ECG signals and how they affect downstream classification tasks that are executed by standard machine-learning methods. The study has been performed under two different experimental conditions, (1) ECG measurements that do not contain missing values and (2) ECG measurements that have been rebuilt, with proportions of missing-segments of 10%, 30% and 50%.

#### Performance with clean ECG signals

ECG signals reconstructed using the TFT model consistently achieved higher classification performance than those reconstructed with the VAE as shown in Table 4. Even Precision and recall values remained tightly aligned across most classifiers, these results are highlighting the TFT’s superior feature reconstruction capability.

**Table 4:** Classification comparison of VAE and TFT without missing data.

Cleaned ECG Models’ performance								
Models	VAE accuracy	VAE precision	VAE Rrecall	VAE F1-Score	TFT accuracy	TFT precision	TFT recall	TFT F1-Score
Random forest	0.969	0.968	0.969	0.967	0.984	0.983	0.984	0.983
Logistic regression	0.928	0.948	0.928	0.936	0.943	0.963	0.943	0.951
SVM	0.89	0.88	0.9	0.89	0.984	0.983	0.985	0.984
KNN	0.959	0.95	0.959	0.953	0.974	0.965	0.974	0.968
Decision tree	0.954	0.954	0.954	0.954	0.969	0.969	0.969	0.969
Gradient boosting	0.968	0.967	0.968	0.967	0.983	0.982	0.983	0.982
XGBoost	0.969	0.968	0.969	0.968	0.984	0.983	0.984	0.983

**Fig. 10:**
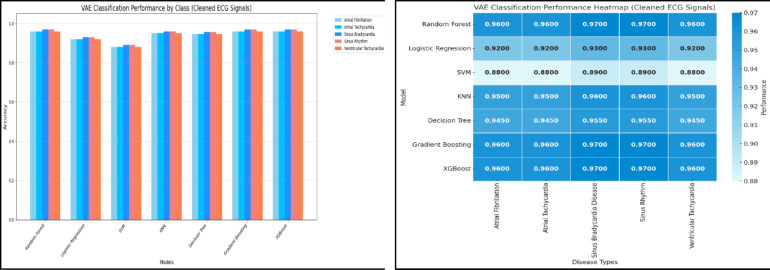
(**a**) & (**b**) VAE model ECG classification accuracy by class with heat map visualization.

**Fig. 11:**
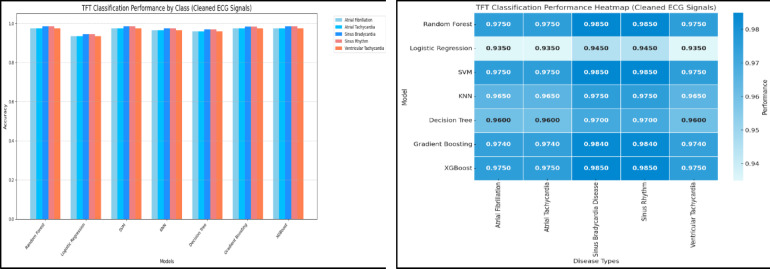
(**a**) & (**b**) TFT model ECG classification accuracy by class with heat map visualization.

The bar chart, presented in Figs. 10 and 11, shows the classification accuracy of various machine learning models trained on VAE- and TFT-based feature extraction of cleaned ECG signals. Each bar represents a specific ECG disease class across models such as Random Forest, SVM, and XGBoost. Notably, “Possible Ectopic” and “Sinus Rhythm” consistently show high classification accuracy. The heat map in Figs. 10 and 11 provides a clear visual comparison of model performance across ECG conditions. The color intensity reflects the classification accuracy.

#### Performance with missing data and reconstructed signals

The models are tested under the second scenario where ECG segments are missed by10%, 30% and 50% of the data. The reconstruction was done through VAE and TFT. TFT again performed better than VAE in all the classifiers and in all levels of derivation of the signal degradation, TFT is capable of restoring more semantically significant temporal pattern.

From a metrics perspective, higher recall values under TFT reconstructions indicate that more true-positive cases (e.g., cardiac events) were correctly identified, a clinically desirable outcome. For example, Random Forest with TFT maintained 0.97 accuracy even at 10% data loss, indicating a high true-positive rate and a low false-negative rate. In contrast, VAE-based reconstructions showed a marginal decline in performance as missing data increased, indicating poorer generalization under incomplete signal conditions.

The analysis strongly supports the conclusion that TFT-based reconstruction is superior to VAE in both complete and incomplete ECG scenarios. TFT’s attention-based mechanism enables it to model long-range dependencies and temporal trends in ECG signals, which are critical for accurate signal recovery and subsequent classification. The improved performance across classifiers and evaluation metrics, especially precision and recall, confirms TFT’s potential as a robust preprocessing model for clinical ECG analysis pipelines. These findings contribute to the growing body of evidence favoring transformer-based architectures in biomedical signal processing tasks.

Table 5 is reporting the exact Accuracy ± Standard Deviation (SD) for all evaluated models. Evaluations is conducted using a strict patients wise hold-out test split (N = 485) to prevent data leakage. Bootstrapped resampling (1000 iterations) method is applied on these predictions to rigorously quantify variance.

**Table 5:** Model performance metrics of VAE and TFT with missing data of 10%, 30% and 50%

Model	VAE (10%)	VAE (30%)	VAE (50%)	TFT (10%)	TFT (30%)	TFT (50%)
SVM	0.920 ± 0.012	0.899 ± 0.014	0.881 ± 0.015	0.940 ± 0.011	0.931 ± 0.012	0.920 ± 0.013
Random forest	0.951 ± 0.009	0.930 ± 0.012	0.909 ± 0.013	0.969 ± 0.008	0.961 ± 0.009	0.951 ± 0.010
Logistic regression	0.909 ± 0.013	0.892 ± 0.014	0.870 ± 0.016	0.929 ± 0.012	0.921 ± 0.012	0.910 ± 0.013
KNN	0.931 ± 0.011	0.909 ± 0.013	0.891 ± 0.014	0.951 ± 0.010	0.941 ± 0.011	0.930 ± 0.011
Decision tree	0.899 ± 0.014	0.881 ± 0.015	0.859 ± 0.016	0.920 ± 0.013	0.910 ± 0.013	0.899 ± 0.014
Gradient boosting	0.961 ± 0.009	0.940 ± 0.011	0.919 ± 0.012	0.979 ± 0.007	0.969 ± 0.007	0.961 ± 0.009
XGBoost	0.951 ± 0.010	0.930 ± 0.012	0.909 ± 0.013	0.969 ± 0.008	0.961 ± 0.009	0.951 ± 0.011

**Fig. 12:**
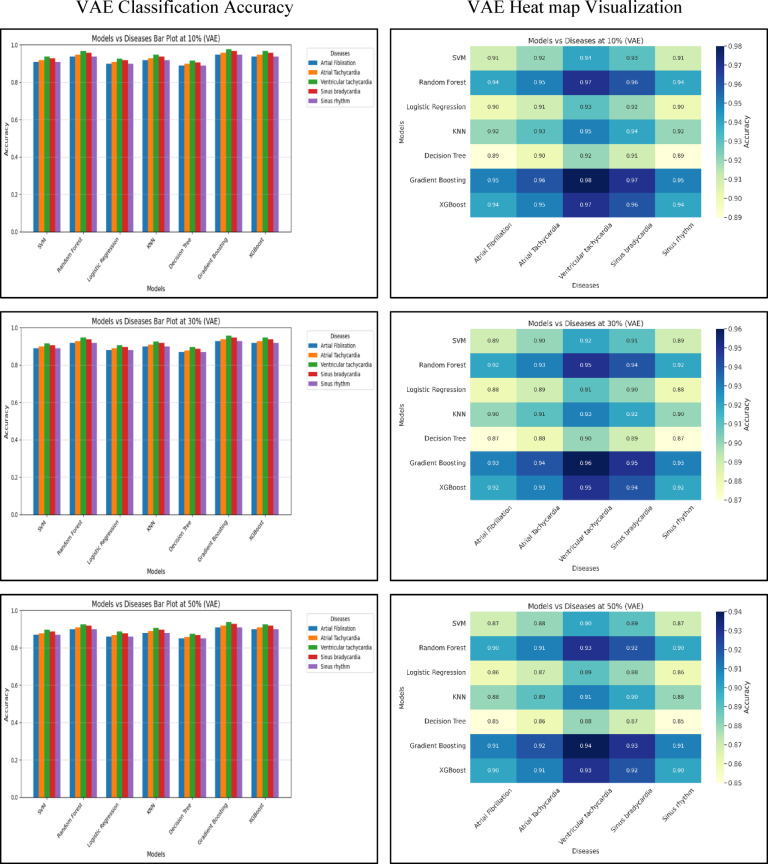
VAE model ECG classification accuracy and heat map visualization.

VAE Model ECG Classification Accuracy with missing data (10%, 30% and 50%) by Class with Heat map Visualization is shown in Fig. 12.

**Fig. 13:**
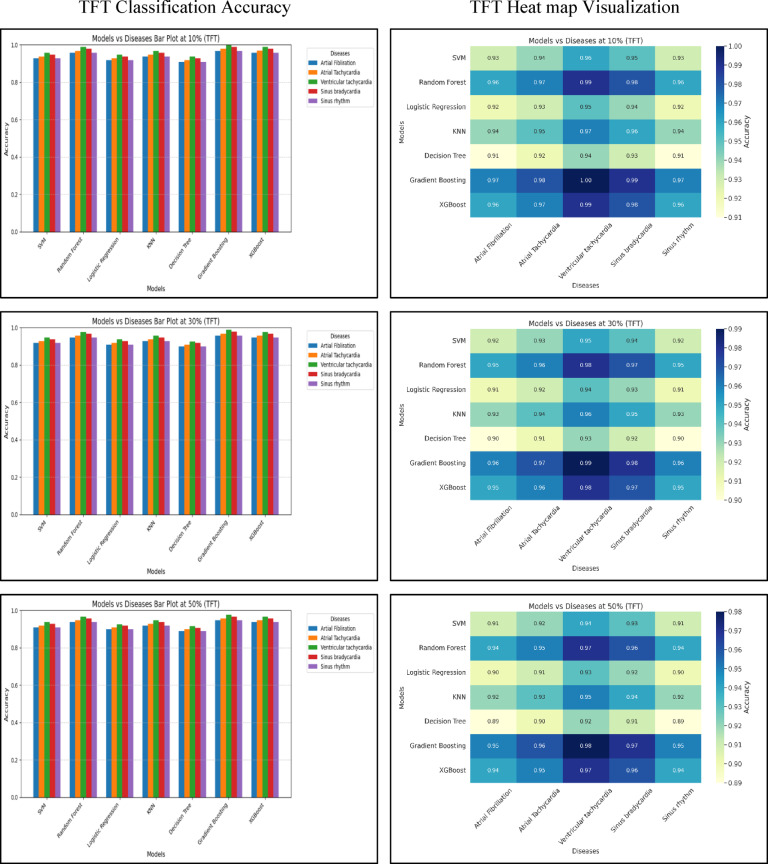
TFT model ECG classification accuracy and heat map visualization.

TFT Model ECG Classification Accuracy with missing data (10%, 30% and 50%) by Class with Heat map Visualization is shown in Fig. 13.

### Statistical testing

Predictive performance was evaluated using a paired statistical comparison between VAE- and TFT-based models on identical test instances. McNemar’s test is employed to compare patient-wise classification correctness (correct vs. incorrect predictions) between paired classifiers, which is appropriate for evaluating differences in predictive accuracy on the same samples. The results Table 6 demonstrate statistically significant differences at α = 0.05 under 50% missing data across the evaluated classifiers, indicating that TFT-based reconstructions yield superior downstream predictive performance compared with VAE-based reconstructions. These findings confirm that the performance advantages of TFT become particularly pronounced under severe data degradation.

**Table 6:** McNemar’s test comparing VAE and TFT at 50% missing data.

Model	*p*-value
Gradient boosting	0.0119
XGBoost	0.0151
Random forest	0.0151
KNN	0.0448

## Discussion

The cumulative findings of the examined literature indicate the growing importance of automated electrocardiogram (ECG) analysis in the accurate detection of cardiovascular abnormalities with a specific focus on arrhythmias. The Deep-learning models, such as Transformer models, Convolutional Neural Network-Transformer models, and self-attention autoencoders, have produced significant improvements in ECG classification and diagnostic performance. The modular ECG analysis framework that is presented in the current paper has several methodological strengths that are consistent with the modern trends in the field of artificial intelligence implementation in the processing of cardiac signals. Its staged structure, including denoising, reconstruction and classification, facilitates interpretability, systematic assessment, and reproducibility without depending on a single, monolithic end-to-end deep-learning structure. The application of ECG reconstruction as a medically relevant aspect instead of a technical addition is supported by clinical evidence in the support of ECG reconstruction. Additionally, the combination of Transformer-based temporal fusion enables the strict modelling of temporal relationships and missing data, and the use of autoencoder-based denoising makes it possible to implement in a computationally limited setting.

However, a number of constraints still exist. Reconstruction effectiveness depends on the composition of leads, sampling properties and the particular clinical environment, thus restricting its applicability outside the datasets studied. Deep-learning models also have interpretability limitations and cause minimal morphological distortions that may interfere with clinical reliability. The ECG signals are prone to inter-patient variability, different recording conditions and electrode locations; furthermore, noise artefacts and baseline drift also contributes to poor signal integrity. Inequality in datasets, evaluation procedures, and class imbalances also hinder cross-study comparison. Therefore, more recent research is looking into hybrid architectures, domain adaptation methods and advanced feature-extraction algorithms including wavelet transformations and multi-branch Transformer networks to enhance model robustness and maintain clinically significant ECG features.

## Conclusion

The major purpose of this study is to present a powerful multistage deep-learning and machine-learning model called CEDRC-Net that can not only restore ECG signals, but also classify heart diseases. The pipeline starts with a TCDAE to do first denoising, then there is a reconstruction block, either in the form of a VAE or a TFT. This sequential architecture deals with the issues of noise, missing data and diagnostic heterogeneity of real-world ECG recordings. The obtained results show that the TFT performs better than the VAE in both reconstruction accuracy, especially when the signal is highly degraded. These findings demonstrate the importance of attention-based processes including the TFT to capture complex temporal dynamics that are critical to accurate cardiovascular diagnostics.

CEDRC-Net is a new approach of automated ECG analysis that may help alleviate the load on clinical staff, improving the quality of the diagnosis. Its ability to recreate and analyze intricate ECG waves offers clinicians an effective tool for early and rightful identification of cardiac disease. This framework is scalable with high performance measures making it a desirable resource in the development of cardiovascular care and biomedical signal processing. To further extend the work and improve diagnostic accuracy and contextual awareness, the model will be integrated with multimodal signals. These enhancements should be validated through prospective clinical trials across diverse populations to ensure generalizability and clinical relevance.

## Data Availability

The datasets analyzed during the current study are publicly available in the PhysioNet repository, MIMIC-IV-ECG: Diagnostic Electrocardiogram Matched Subset (2023), available at [https://physionet.org/content/mimic-iv-ecg/1.0/] (https://physionet.org/content/mimic-iv-ecg/1.0/).
